# Continued breastfeeding and work: scenario of maternal persistence and resilience

**DOI:** 10.1590/0034-7167-2022-0191

**Published:** 2023-01-30

**Authors:** Isília Aparecida Silva, Carla Marins Silva, Elisiany Mello Costa, Micheli de Jesus Ferreira, Erika de Sá Vieira Abuchaim

**Affiliations:** I Universidade de São Paulo. São Paulo, São Paulo, Brazil; II Universidade Federal de São Paulo. São Paulo, São Paulo, Brazil

**Keywords:** Breast Feeding, Work, Resilience, Psychological, Women, Qualitative Research., Lactancia Materna, Trabajo, Resiliencia Psicológica, Mujeres, Investigación Cualitativa., Aleitamento Materno, Trabalho, Resiliência Psicológica, Mulheres, Pesquisa Qualitativa.

## Abstract

**Objectives:**

to understand the challenges in mothers’ daily life and strategies adopted to reconcile activities outside the home and continued breastfeeding.

**Methods:**

a cross-sectional, qualitative study. Theoretical-methodological assumptions were discursive practices and production of meanings in everyday life. Participants were 22 women from a specific social media group who had breastfed at least one child for >7 months. Data were collected between November 2020 and March 2021.

**Results:**

themes: Around the world of activities outside the home; Work environment: routines, opportunities and difficulties to maintain breastfeeding.

**Final Considerations:**

women’s experiences reveal a daily life with difficulties in reconciling the desire to breastfeed and the work scenario. Support network and adaptation to children’s food routine were strategies adopted to minimize risks of weaning. The results show the need to consolidate policies to encourage continued breastfeeding in the labor market.

## RESUMO

**Objetivos:**

compreender os desafios do cotidiano materno e as estratégias adotadas para conciliação entre as atividades fora do lar e a amamentação continuada.

**Métodos:**

estudo transversal, qualitativo. Pressupostos teórico-metodológicos foram: práticas discursivas e produção de sentidos no cotidiano. Participaram 22 mulheres, de um grupo de mídia social específico, que amamentaram pelo menos um filho por > sete meses. Dados foram coletados entre novembro de 2020 e março de 2021.

**Resultados:**

temas: A volta ao mundo das atividades fora do lar; Ambiente no trabalho: rotinas, oportunidades e dificuldades para manter a amamentação.

**Considerações Finais:**

a vivência das mulheres revela um cotidiano com dificuldades de conciliar o desejo de amamentar e o cenário laboral. Rede de apoio e a adaptação na rotina alimentar da criança foram estratégias adotadas para minimizar os riscos de desmame. Os resultados mostram a necessidade de consolidação das políticas de incentivo ao aleitamento continuado no mercado de trabalho.

## RESUMEN

**Objetivos:**

comprender los desafíos del cotidiano de la madre y las estrategias adoptadas para conciliar las actividades fuera del hogar y la continuación de la lactancia materna.

**Métodos:**

estudio transversal, cualitativo. Los supuestos teórico-metodológicos fueron: prácticas discursivas y producción de sentidos en la vida cotidiana. Las participantes fueron 22 mujeres, de un grupo específico de redes sociales, que habían amamantado al menos a un niño durante más de 7 meses. Los datos se recopilaron entre noviembre de 2020 y marzo de 2021.

**Resultados:**

temas: En torno al mundo de las actividades fuera del hogar; Ambiente de trabajo: rutinas, oportunidades y dificultades para mantener la lactancia materna.

**Consideraciones Finales:**

la experiencia de las mujeres revela un cotidiano con dificultades para conciliar el deseo de amamantar y el escenario laboral. La red de apoyo y la adaptación a la rutina alimentaria del niño fueron estrategias adoptadas para minimizar los riesgos del destete. Los resultados muestran la necesidad de consolidar políticas para incentivar la continuación de la lactancia materna en el mercado laboral.

## INTRODUCTION

Breastfeeding has occupied a significant place in the political and scientific agendas, considered one of the best investments of society in the development of human potential, capable of contributing to the improvement of the physical, cognitive and social capacity of children in adult life^(1)^. The universal practice of breastfeeding up to 2 years or more is able to prevent 12% of all deaths of children under 24 months and save US$300 billion annually^(2)^.

The World Health Organization (WHO) recommends exclusive breastfeeding up to six months of life and continued breastfeeding up to 24 months or more, not indicating when total weaning should occur^(1)^. The prevalence of breastfeeding in children up to 12 months is 68%, and up to 24 months, it does not exceed 44% of children worldwide^(3)^. In the last 34 years, Brazil presented a 22.7 and 23.5-fold increase in breastfeeding of children up to 12 and 24 months, respectively^(4)^.

Continued breastfeeding has not received the same attention from promotion, incentive and support programs as the practice of exclusive breastfeeding. The same limitation is observed among scientific studies, which have little explored this practice and its protective and risk factors, in the national and international population^(5)^.

Despite its importance, women face difficulties in maintaining breastfeeding after the child is six months old, and activities outside the home, especially work, are one of the most challenging elements for continuity of this practice^(6)^. In the last 20 years, the participation of women in the labor market has increased, reaching 48% in the current decade^(7)^. The adoption of strategies, such as the guarantee of paid maternity leave and the legal right of the nursing mother to enjoy, in the first six months after childbirth, two special breaks (30 minutes each), during the working day (CLT), has had a positive effect on increasing exclusive breastfeeding rates, but does not impact continued breastfeeding rates^(1)^.

In this scenario, it is important to reflect on the dissonance between the demand for improving breastfeeding rates, according to the WHO recommendations, and the social and cultural basis offered to women, so that the practice of continued breastfeeding, and here also called prolonged, be made possible^(1,3)^.

It is necessary to investigate, in greater depth, the maternal challenges in maintaining continued breastfeeding, reconciling their activities and living in the social environment without constraints.

## OBJECTIVES

To understand the challenges in mothers’ daily life and strategies adopted to reconcile activities outside the home and continued breastfeeding.

## METHODS

### Ethical aspects

The research was submitted to analysis of by the Research Ethics Committee at the School of Nursing of the *Universidade de São Paulo*, being approved under CAAE (*Certificado de Apresentação para Apreciação* Ética - Certificate of Presentation for Ethical Consideration). To ensure anonymity, the statements are identified with the letter E, followed by the Arabic number, which corresponds to the order in which the sessions were held.

### Study design

This is a cross-sectional, qualitative study, with the theoretical-methodological assumptions: discursive practices and production of meanings in everyday life^(8)^. “Discursive practices represent the language used in everyday life, they are the ways in which people, through language, produce meanings and position themselves in everyday social relationships”^(8)^.

The meanings are produced by the interaction between people, being essentially a dialogic and discursive social practice, molded from linguistic repertoires, created in three historical times: “Long Time is the domain of the construction of cultural contents that were part of the discourses of a given time (...); Lived Time is the time of resignification of these historical contents from the processes of socialization (...); Short Time is the time of dialogic interanimation and the dynamics of the production of meanings”^(8)^.

### Population

Defined by data saturation, the sample consisted of 22 women aged 18 years or older, having breastfed at least one child for more than seven months, being a member of a specific social media group, selected by the researcher, regardless of the time of participation. Among the multiparous women, the relationship with the child with longer breastfeeding time was considered for this study.

### Search setting

Research carried out in a virtual environment, together with a specific social media group, a meeting place for women seeking support and guidance on the breastfeeding process.

### Identification and access to participants

The coordinators of social media group invited the members to join the study through an electronic invitation posted in the group. As the women registered, they were contacted by the researcher in charge via e-mail, which forwarded the link to Google Forms for access to the Informed Consent Form (ICF), clarifying the objectives, inclusion criteria and procedures for conducting the research. Those who agreed to participate in the study registered their consent in the virtual document itself.

Data collection was carried out by the senior researcher responsible for the study, between November 2020 and March 2021, via Google Meet. The sessions were carried out individually, starting with the collection of objective data characterizing the mother, child, family, obstetric and breastfeeding history. Next, the open-ended, in-depth interview, from the perspective of discursive practices, began with the guiding question: could you tell me how it was or how has it been your experience of breastfeeding your child for a long time? All sessions were recorded, image and sound, and, later, the verbal content was transcribed in full. All material was stored in a virtual environment, Google Drive, protected by password, known only by the main researcher.

### Data analysis

Quantitative data were organized into tables and analyzed descriptively and percentageally. The process of analysis and interpretation of qualitative data followed the methodological proposition of Spink^(8)^: reading and global identification of the researcher’s and participants’ statements, systematization of the analysis process with the construction of maps of association of ideas and definition of thematic categories.

## RESULTS

### Introducing women, their children, and their breastfeeding stories

Twenty-four women accepted to be part of the research, however two were unable to reconcile the times for the session, even after several scheduling attempts. The study included 22 women with a mean age of 38 years; 86% were in a stable marital union; 100% had a higher education level; 40% had a *stricto sensu* graduate degree; and 45% were primiparous. The investigated ones considered that, among the activities carried out outside the home, the work ones were those with the greatest impact on continued breastfeeding.

Chart 1 shows the characteristics related to the duration of breastfeeding in the first and last child, as well as the work situation and duration of maternity leave in the same periods.

**Chart 1:** Interviewee characteristics in terms of parity, duration of breastfeeding, employment status and time of maternity leave at the birth of the first and last child, São Paulo, São Paulo, Brazil, 2021

Interviewee	Parity	First child			Last child		
		BF (months)	Employment relationship	ML (months)	BF (months)	Employment relationship	ML (months)
E1	Multiparous	24	Yes	6	3	Yes	6
E2	Multiparous	32	Yes	6	23	Yes	6
E3	Primiparous	28	Yes	6	-	-	-
E4	Multiparous	12	Yes	4	30	Yes	4
E5	Multiparous	43	Yes	4	9	-	0
E6	Multiparous	31	Yes	4	5	Yes	4
E7	Multiparous	19	Yes	4	36	No	0
E8	Primiparous	20	Yes	6	-	-	-
E9	Primiparous	40	Yes	6	-	-	-
E10	Primiparous	37	Yes	4	-	-	-
E11	Primiparous	31	Yes	4	-	-	-
E12	Primiparous	40	-	0	-	-	-
E13	Primiparous	40	Yes	6	-	-	-
E14	Primiparous	30	No	4	-	-	-
E15	Multiparous	42	No	4	35	No	4
E16	Primiparous	44	Yes	6	-	-	-
E17	Multiparous	46	Yes	4	35	Yes	4
E18	Multiparous	38	Yes	6	34	Yes	4
E19	Multiparous	48	No	0	30	No	0
E20	Primiparous	25	Yes	4	-	-	-
E21	Multiparous	19	Yes	6	24	Yes	6
E22	Multiparous	43	No	4	33	No	4

*ML - maternity leave; BF - breastfeeding.*

Considering parity among the primiparous, the average breastfeeding was 33 months and 15 days of a child’s life, and two women were still breastfeeding at the time of the interview. With regard to multiparous women, the average was 33 months and 03 days and 28 months and 27 days for the first and last child, respectively. The two nursing mothers with children under seven months of age were not considered for this calculation.

### Persistence and resilience in maintaining breastfeeding indefinitely

The first six months of children’s life involved overcoming physical and emotional limits, establishing a rhythm and empowering the practice of breastfeeding and, often, a proof of resistance in the face of contrary and contradictory opinions, coming from their social context and from professionals who watch them. The end of this period includes, for most women, a new element, which is the return to activities outside the home, especially professionals, since few have the opportunity or condition to stay at home with full dedication to their children.

Some women, even before the baby is six months old, begin to plan their (re)adaptation to the environment outside the home, aiming to reconcile breastfeeding and professional activities. The first step is how to organize a routine and an environment conducive to lactation and breastfeeding maintenance, whether in the work environment arrangements, for expressing and storing breast milk, or in domestic arrangements, to guarantee a caregiver and the supply of their milk, stored for offering to the baby when they are absent. With the measures overcome, a new confrontation is present with the passage of lactation time, exposing it to judgment of the actors in surroundings, due to the fact that it is breastfeeding a child above the socially recommended age for the baby.

#### T.1. Around the world of activities outside the home

The return to work, school or other activities outside the home throws the woman into the challenge of maintaining breastfeeding and, therefore, encouraging lactation, during long periods away from the child.

##### T.1.1. The saga to maintain breastfeeding: need for a support and adaptation network

Despite this being the wish of the majority, not all women have the option or condition of not working and voluntarily staying at home to take care of their children. Thus, the identification of a support network is necessary for the woman to be sure that her milk will be offered to her child in an adequate way and without causing interference or sucking addictions, whether offered at school, day care or at home by people trained and equipped for this purpose. For many caregivers, it is difficult to adapt to the new pattern of offering breast milk, in cups or spoons, which often makes it difficult to find people who perform the task to the mother’s satisfaction.

[...] *I went to design a schedule of activities and a support network for who would be with my daughter, who would offer the milk in my absence. But I had an easy time even for that, despite a lot of fear and anxiety about how she was going to look, how breastfeeding was going to continue. But I feel like I took all the care to make it go as smoothly as possible.* [...] *I took my mother to a breastfeeding workshop,* [...] *we saw a lecture together and it was aimed at caregivers* [...] *and then he showed my mother in the kitchen of the clinic how to heat milk in water for my mother, how to put it in the cup, how to offer it. Every baby too* [...]. (E0)

Some situations are more favorable to women and babies, when there is a day care center or nursery at or close to the work place, which facilitates breastfeeding during the workday or at established times, but does not break the breastfeeding pace. For some women, depending on the context of work or school activity, it is possible to take the child with them and breastfeed, or even implement an activity dynamic that allows breastfeeding before leaving home and when returning home.

*I think I was very lucky. I was very privileged, because he stayed in the nursery of the company my husband worked for and it was 2 km from where I was. We stopped at the office where my husband worked, I had access to the living room* [...] *the nursery. I would go in there, breastfeed, leave him in his little room, take the car and go to work. At lunchtime, I came back. In between, right? Come in* [...] *in that break in the morning, I was milking.* (E8)

##### T.1.2. Combining breast, expressed milk and food

The initial goal to maintain breastfeeding is to provide as much milk as possible to replace the feedings at times when the mother is away from the child. For this purpose, the condition of expressing the milk before returning to work to leave a stock ready to be used would be ideal, but it is not always possible. Continuity of breastfeeding process requires that the milk be expressed daily, for children’s consumption.

*So, I ended up taking five and a half months off. When I returned* [...] *from the first two, I didn’t get to make a milk bank, but I took it daily at work and took it home and they suckled that day’s milk. In case it was missing, I supplemented it with formula* [...] *the third and fourth, I made a bench. I froze in advance. Before going back to work, I already made a nice bank* [...] *storage of milk at home* [...]. (E3)

Some women are able to delay the time to resume outdoor activities by adding vacation time or special leave. Thus, when they return to work, the child starts to eat other foods, and some of the meals are breastfeeding, when the nursing mother is at home. Even when there is a predominance of other foods and expressed breast milk is not the children’s main meal, milk expression is still necessary, even if it is for maternal relief or to maintain lactation stimulation to have milk in the feedings made in the periods that is with children.

*So, I work on the street all the time. And I already had, for me, that I was going to get milk. One: because the quantity bothered me. Two: to offer to my son, right? So, he stayed until he was practically two years old* [...] *from those seven months until he was two years old, he stayed with my mother. And then I would breastfeed at home before going to my mother. Then, at my mother’s* [...] *I didn’t introduce breakfast. He was based on fruits in the morning, he ate lunch and then, in the afternoon, I always left the milk for a feeding, which was this milk that I had frozen at work. Then I took him* [...] *he suckled, and then he practically didn’t let go of my breast.* (E11)

#### T.2. Work environment: routines, opportunities and difficulties to maintain breastfeeding

The practice of expressing milk by lactating women can be experienced in very different ways in their work environment. Breast milk expression and storage demand a place and minimum conditions of privacy, hygiene and reception by co-workers. Support can come from the institution itself, by offering relevant logistics, and from the activity arrangements, which can be made more flexible to give women the opportunity to carry out, in particular, milk expression in a relevant and efficient way. However, progressively, the work routine pace absorbs the nursing mothers’ time, reduces the opportunities for expression, and co-workers start to resent it and interpret it as a privilege for women to be absent during periods of attention to lactation.

##### T.2.1. Adequate location, flexible schedules and partnership with co-workers

Women need to feel accepted in their lactation status and in their desire to continue lactation. This will require a partnership with co-workers so that they have the condition to be absent from the work place at regular periods, in addition to an appropriate place and the use of utensils and equipment for milk expression and storage.

*Also, what made it easier for me is that, at my work, I had a private room where I could express the milk, store it in the freezer itself - which was only for the mothers who expressed the milk - and then, at the end of the day, I took my milk there to school. So, it made my logistics a lot easier, my routine of being able to breastfeed my daughter.* (E4)

*The company was super open. Even after six months, right? Because, at the time, I didn’t even have a CLT relationship. My hire was through my company. Even so, the company gave me the benefit of maternity leave and never complained about the breaks I took to milk, even after six months, right? Because the labor legislation requires, so to speak, that the company allows the mother to take milking breaks up to six months. I continued* [...]. *My eldest, as she went to school* [...]. *And then we got into a super rhythm* [...]. *I sent milk to her, to school beyond a year.* (E13)

Solidarity and partnership of women in the work environment appear as social elements that support the goal of prolonged breastfeeding of nursing mothers. The support network is shown to be extended in places where other women have gone through or are going through the same type of experience. They are welcoming and protective voices that show the production of meanings of time lived in the work environment of these women as a positive inauguration where this experience did not yet exist, but that a reiterated collective movement, of continuity of care from other women who have gone through the same situation, can build the receptivity of all to provide the experience of women in maintaining their lactation and breastfeeding.

*And I also had the support of a co-worker. When I went back to work, with the first daughter, she was going back to work. She had twins, then she arrived at the office where we work and spoke to the boss. She asked for a meeting room to express the milk and I don’t think I would have asked for myself, but as she asked and she got it, she let me know. So, I already used that meeting room in the first one and I already used it in the second one. It was great, because I think that if I was in a shared bathroom, I wouldn’t take it out, you know? So long. So, I think that was it too. This help from her helped me to continue expressing milk to give to her and to him.* (E19)

##### T.2.2. Work routine competes with breastfeeding

Over time, the demand and dynamics of work are not always maintained with the minimum desirable conditions of flexibility and acceptance so that the expression routine is maintained, progressively imposing limits on the freedom of women to be absent from work to alleviate a lactating breast. Gradually, the work routine imposes itself, limits and restricts the opportunities for women to maintain lactation, causing frustration, resentment with the work context and provoking abandonment of maintaining a productive activity of milk.

*But I never thought it was a problem I had to solve. I thought it was a problem that work had to solve. What do you mean? It’s not a normal thing. Sometimes I walked into meetings that lasted four, five hours. It was unbearable for me. Unbearable. A normal thing before pregnancy and such, but it started to get unbearable because of it, having to go out and such. That was an obstacle.* (E18)

*I stopped milking he was almost a year old, because milking, after a while, I got very tired of continuing to milk at work* [...] *because of the dynamics. That’s what work is, there’s a lot of demand. I couldn’t stop anymore, and then you saw the milk supply decreasing and that made me very stressed.* (E1)

##### T.2.3. Not everyone helps: the work context tolerance limit

Even in professional contexts where there are good logistical and reception conditions for nursing mother, interpersonal relationships may not be compatible with the structural environment and the facilitating operational environment. The lack of collaboration and empathy for co-workers at work and the way in which the questions are asked cause embarrassment to the nursing mother.

*But I had a problem, like changing the temperature of the fridge, because they thought it was freezing too much, and then I lost the milk because it had frozen and thawed* [...] *I had problems in that sense and some comments that we get to know there, right? One or the other commented “But she stops twice a day, plus lunch, to go to the meeting room”.* (E8)

*There was the question of when I got back to work, which was pretty boring. It was about the respect of professionals to understand that you need to milk, right? That was very difficult, because, in my midst, I had never seen another woman breastfeed. None breastfed. In my work environment, it wasn’t a thing either* [...] *I had never seen a woman saying, “Excuse me, I’m going to milk”. Didn’t have it. I don’t know. I think people really give bottles, so it was kind of an uncomfortable thing to do* [...] *to have to go out* [...]. (E18)

*Of course, at work, there was a questioning, because there is in the CLT that lactating women cannot go to the internship field and* [...] *I was asked about being breastfeeding or not.* [...] *then, I should write a letter declaring myself breastfeeding. My daughter at that time was under 9 months.* [...] *the HR* [...] *asked about the need to feed my baby with breast milk* [...] *and they asked for a medical report from the pediatrician, right?* [...] *the HR asked until when a woman could be released for being breastfeeding, until when she could stay away* [...] *and then there was a certain conflict: what do you mean my right to breastfeed has to be questioned or does it need to be a paper, a medical report?* (E0)

##### T.2.4. Inappropriate place/no conditions for extracting and storing breast milk

The lack of private and appropriate places for this purpose makes the nursing mother use improvised places. Usually, she uses the toilets or some other space that, although it has another destination, can give her moments of privacy. The lack of minimally adequate conditions can make the milk expression process very difficult or unfeasible.

*But I didn’t milk in a specific place, tidy for me for that. The space that was given to me so that I could milk in privacy* [...] *I was still lucky, because there was no bathroom. There was a room where the cleaning, maintenance staff* [...] *every now and then the maintenance guy or the security guard came in. So, in those moments that I was going to milk in this room, I could lock it. I was allowed to lock the room from the inside, and then if any of them needed to come in, they would knock on the door. I covered myself, opened it and everything was fine. Then, there was a whole change of space in the company and this room would no longer be available to me, and I continued milking in the server room, which was a cubicle like that. And then there was a chair and an outlet that was free there and I would enter this little room, lock the door and stay there.* (E13)

*Because, at work, I found it very unhygienic. I had no conditions. Every hour I was in a bathroom. I don’t know*[...] *I was kind of tense. How would I store it? That thing* [...] *had a thousand schemes, right? I don’t know* [...] *I didn’t know* [...] *then it’s going to stay in my bag for ten hours and then I put it in the fridge? It’s kind of weird, right? I don’t know* [...] *I couldn’t.* (E18)

## DISCUSSION

The understanding of the production of meanings in the mother’s daily life, about the practice of continued breastfeeding, shows that the return to activities outside the home, especially to work, constitutes a great challenge to prolonged breastfeeding maintenance and proof of maternal resilience. Despite the scarcity of studies on the relationship between maternal work and the duration of prolonged breastfeeding, the findings of the present study reveal that the experience of maintaining breastfeeding for an even longer period of time, meeting official health recommendations, also needs to be incorporated into the public agenda and from the perspective of society, whether for children’s physical and emotional health, or for respect for women who choose this style of eating and relationship with their children.

Maternal work outside the home is an important barrier to maintaining lactation and breastfeeding, revealing an unquestionable factor in promoting weaning^(9-11)^. There are laws to protect the nursing mother in Brazil, however the separation between mother and child has a direct impact on the routine of breastfeeding and, consequently, on lactation maintenance^(9-10)^.

With the end of maternity leave, women seek ways to maintain breastfeeding. Finding caregivers, extracting and storing milk and offering it to the child are elements of an organized planning to guarantee milk storage and supply. Each woman, depending on her context, in addition to the milk expressed and offered in different ways, organizes a routine that also includes breastfeeding moments when she is with the child^(5)^.

In this scenario, the support network formed by family, friends and, especially, by partners plays an extremely important role in supporting and understanding the decision-making of this working woman, aiming at success in the desire and goal of breastfeeding^(9)^.

The work environment must offer objective conditions for the expression, packaging and refrigeration of breast milk. It also implies a social space in which nursing mothers find welcoming and comprehensive solidarity to practice lactation maintenance, or breastfeeding schedules, when there are day care centers in place^(10)^. A work environment that recognizes, values and supports breastfeeding promotes greater commitment and lower absenteeism rates among its workers^(12)^.

The women in this study had different experiences regarding the conditions to maintain lactation, from day care centers incorporated into the company, private environments and appropriate equipment for milk expression and storage, and others were not so lucky, having to improvise locations and equipment for this purpose. However, in addition to material conditions, the interrelationships of everyday life showed that intolerance to flexible schedules, such as the possibility of completing their workday in advance after completing daily tasks or choosing more convenient work shifts and sporadic maternal absences for breastfeeding or milk expression and the resumption of work dynamics pace, can hinder the possibilities of continuing this process for a long time^(9,11)^.

In the words of these women, work ends up causing overload and fatigue. The lack of time in the work routine prevents breast milk expression, favoring the gradual reduction of milk production, increasing the risk of breast complications and generating frustration and abandonment in maintaining lactation^(11,13)^. The need for daily expression to guarantee the reserve of milk to be offered to children in maternal absence, a condition that seems to be common in different scenarios, is another source of stress and anxiety, in addition to the maternal sleep deprivation experienced since the beginning of breastfeeding^(9,11)^. The breastfeeding routine starts to be performed when she is with the child in morning, afternoon and night feedings, in an attempt to maintain breastfeeding for as long as possible or as planned.

Nursing mothers perceive attitudes of judgment and disapproval, for maintaining breastfeeding for longer than expected by society, around six to seven months. The feeling of intolerance and disapproval increases. The older the child, it is pointed out that the approval and encouragement of breastfeeding have an expiration date for the first six months of the child^(11,14-15)^.

Despite having the determination to continue breastfeeding and having support and knowledge, the nursing mother reports facing difficult situations, which they have to overcome. When she decides not to give up, she does not always do so just for herself, but usually because she finds benefits for herself or her child, or for both, even if in different dimensions.

Thus, for lactating workers to be able to breastfeed for two years or more, with exclusive breastfeeding for the first six months, it is essential that, after maternity leave, they have the support of employers. In addition to this support, it is necessary for women to have a social support network, facilitating the process, by taking children to the workplace, for instance, and other aspects that contribute to encouraging continuity of breastfeeding. Mothers who enjoy the emotional and practical support of the family tend to find a balance between work and breastfeeding^(9,11,16)^.

A transparent breastfeeding-friendly workplace policy and legislation that incorporates local labor legislation and is well communicated to the workforce can help women plan for continued breastfeeding^(17)^. However, institutional policy, as well as labor legislation, by itself, does not guarantee the necessary changes for breastfeeding reception and continuity. It is essential to carry out ongoing support approaches and effective communication between women, their managers and all actors around nursing mothers to clarify the possibilities to meet expectations about returning to work and maintaining breastfeeding for as long as it makes sense for mothers and children. Broader dissemination strategies, better information and publicity of these policies are also needed so that there are workplaces favorable to prolonged breastfeeding^(16)^. A collective effort is needed in the social context of nursing mothers, in order to open spaces for the re-signification of observed meanings of breastfeeding duration limitation and a new identity for long-term breastfeeding nursing mothers.

### Study limitations and contributions to nursing

Most women had a partner, high level of education, previous pregnancies and previous and current experience of prolonged breastfeeding, which does not guarantee the representation of social diversity. This situation raises the need to replicate the study in other social groups, to understand the reality experienced by workers, on the world stage, who aim for continued breastfeeding.

## FINAL CONSIDERATIONS

The experience of nursing mothers who work in their real challenges and strategies necessary to reconcile and achieve success in continued breastfeeding reveals that daily work activities and the misunderstanding of most co-workers can bring difficulties in the attempt to breastfeed indefinitely, leading women to seek ways, even if solitary, to maintain lactation. On the other hand, the search for a support network and adaptation to children’s food routine are strategies adopted to minimize the risks of weaning. Such an understanding sheds light on useful and necessary referrals to help women who wish to reconcile work activities with breastfeeding their children for the desired time. Thus, an effort by society is necessary to ensure compliance with official breastfeeding recommendations for two years or more, since it is evident that current initiatives partially guarantee the maintenance of this practice.
